# Linking tick and wildlife host distributions to map risk of tick-borne diseases

**DOI:** 10.1186/s13071-025-07096-0

**Published:** 2025-11-19

**Authors:** José-María García-Carrasco, David W. Crowder, Karen C. Poh, Juan Mosqueda, Massaro W. Ueti, Javier Gutierrez-Illan

**Affiliations:** 1 Department of Entomology, Washington State University, Pullman, WA 99164 USA; 2 Animal Disease Research Unit, USDA-ARS, Pullman, WA 99164 USA; 3 Universidad Autónoma de Querétaro, Avenida de las Ciencias S/N, Juriquilla, C.P. 76230 Querétaro, Mexico

**Keywords:** Biodiversity, Disease ecology, Livestock, One health, Tick, Vector-borne disease, Veterinary

## Abstract

**Background:**

Tick-borne pathogens threaten livestock worldwide, causing diseases such as anaplasmosis, babesiosis, heartwater and theileriosis in cattle. The epidemiology of each disease is complex, with multiple tick and/or host species interacting across variable environments, and disease risk has not been fully assessed. Here, we used a One Health approach that integrates ecological, livestock and wildlife factors to identify areas with potential risk of tick-borne pathogen circulation.

**Methods:**

Using ecological data on tick and host distributions alongside environmental and anthropogenic variables, we modeled the potential ranges of the four tick-borne diseases in North America and the Caribbean based on tick distributions. By integrating these disease-specific models, we generated comprehensive risk maps highlighting cattle exposure hotspots. We further evaluated how ungulate host communities shape the spatial patterns of tick-borne disease risk.

**Results:**

Livestock operations in the central and eastern USA and in southern Mexico are the most vulnerable to tick-borne pathogens. Models demonstrated higher performance when incorporating ungulate host distribution, enabling us to identify the key ungulate species influencing tick distributions and the risk of tick-borne diseases.

**Conclusions:**

Areas with greater ungulate diversity had greater tick diversity, further demonstrating the role of host community structure in shaping the dynamics of tick-borne pathogens. Identifying regions of North America with high exposure to tick-borne pathogens by assessing complex interactions between pathogens, hosts and vectors can aid in developing control strategies to safeguard cattle health.

**Graphical Abstract:**

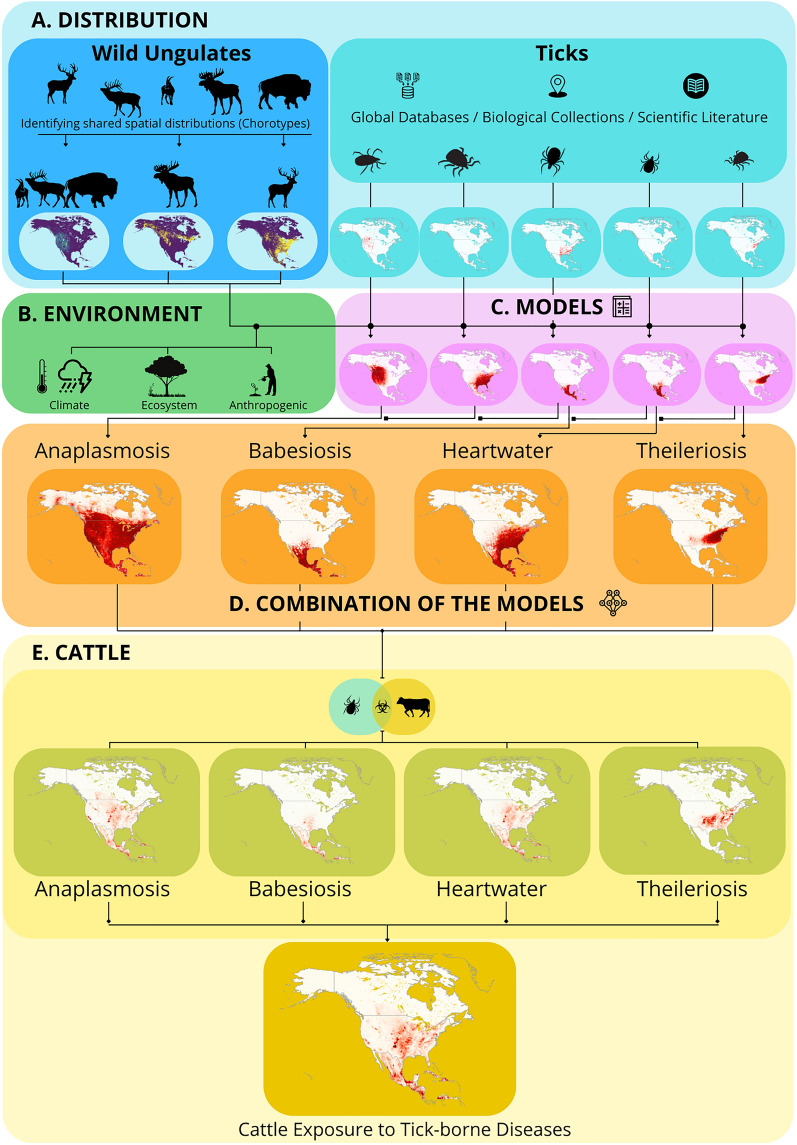

**Supplementary Information:**

The online version contains supplementary material available at 10.1186/s13071-025-07096-0.

## Background

Vector-borne pathogens are a global concern, with ticks transmitting more types of pathogens to humans and livestock than any other arthropod vector [1]. Tick-borne pathogens can affect livestock by reducing milk production, causing weight loss and increasing mortality [1, 2]. Management of tick-borne pathogens often involves control of tick vectors, given that breeding animals for genetic resistance to tick-borne pathogens is difficult [1]. However, it is challenging to predict the prevalence of tick-borne pathogens given the complex interactions among pathogens, vectors, humans, domesticated animals and wild hosts that govern transmission across variable environments [3, 4]. These intricate dynamics necessitate a comprehensive One Health approach to assess the underlying biotic and abiotic factors mediating the spread of tick-borne diseases.

Tick-borne livestock diseases are caused by many pathogens. Among bacterial diseases of concern, anaplasmosis (*Anaplasma marginale*) is present in North America and the Caribbean, while heartwater (*Ehrlichia ruminantium*) is found in the Caribbean [2, 5]. In contrast to the bacterial diseases, babesiosis and theileriosis are caused by protozoa in the genera *Babesia* and *Theileria*, respectively [6, 7]. *Anaplasma marginale* is transmitted by multiple tick species across four genera [2], while *Babesia* is transmitted by two species of cattle fever ticks (genus *Rhipicephalus*) [7]. Yet, emergence of theileriosis in North America has been associated with a single vector, the Asian longhorned tick (*Haemaphysalis longicornis*) [8], and heartwater is only transmitted in the Caribbean by the tropical bont tick (*Amblyomma variegatum*) [9]. This diversity of vectors and pathogens, which interact in unique ways, makes prediction of tick-borne pathogen transmission a challenge requiring an integrated approach [10].

The geographical distribution of tick-borne diseases is often linked to the abundance of ticks across space, which in turn is affected by abiotic factors such as temperature and precipitation [1, 10]. Most ticks can also use a diversity of wild species as hosts, and the competence of these hosts as tick and pathogen reservoirs can vary, such that variations in host communities affect tick and pathogen distributions [11]. For example, while early studies suggested host diversity may lead to ‘dilution effects’ that slow pathogen spread, empirical evidence for this effect is mixed [11]. Yet, while the prevalence of tick-borne pathogens has been mapped in hundreds of studies from wild, domesticated and human hosts [12], few spatial models of ticks or pathogens consider host dynamics over space [13]. This is a major knowledge gap, and a more integrated approach to modeling tick-borne pathogens is needed [14, 15].

In this study, we aimed to improve predictions of tick-borne disease risk in North American livestock using an integrated modeling approach that incorporates environmental, vector- and host-related factors. By combining a One Health perspective with species distribution modeling, we sought to identify the ecological drivers of tick distribution and highlight areas of elevated risk for four major livestock diseases. Our goal is to support early warning systems and inform targeted surveillance efforts by identifying regions where environmental conditions, tick vectors and host communities converge to increase the risk of pathogen transmission to livestock.

## Methods

### Study extent and tick-borne pathogens analyzed

Our study considered tick-borne pathogens affecting livestock in North America, including Canada, the USA, Mexico and the Caribbean. We also considered the Central American countries of Guatemala, Belize, El Salvador, Honduras, Nicaragua and Costa Rica to account for influences of neighboring territories. To account for the size disparity of administrative units across and within countries, we created a standardized hexagonal grid for the study area. Each hexagon of the grid has an area of approximately 500 km^2^, facilitating unbiased comparisons across the study area without using administrative boundaries [16]. For model development, all tick records within a unit were aggregated and treated as a single presence to mitigate spatial autocorrelation and sampling bias.

We focused on four major livestock diseases: (i) anaplasmosis (*Anaplasma marginale*), (ii) babesiosis (*Babesia bovis* and *Babesia bigemina*), (iii) heartwater (*Ehrlichia ruminantium*) and (iv) theileriosis (*Theileria orientalis* Ikeda). These pathogens are transmitted by 13 tick species across five genera (*Amblyomma*, *Dermacentor*, *Haemaphysalis*, *Ixodes* and *Rhipicephalus*).

### Tick and ungulate wildlife host databases

To model the potential risk of each pathogen, we used the distribution of their vectors as a proxy. Tick occurrence data were gathered from the Global Biodiversity Information Facility (GBIF), VectorMap, iNaturalist and collections at Universidad Nacional Autonoma de Mexico (Mexico City, Mexico). We also searched on Web of Science, Scopus and Google Scholar using keywords specific to the ticks and countries of interest. The search included scientific articles, theses and reports that provided occurrence data for the tick species studied. We focused on 13 tick species across five genera (*Amblyomma*, *Dermacentor*, *Haemaphysalis*, *Ixodes* and *Rhipicephalus*) known to be involved in the transmission of the four pathogens of interest in our study (Table 1). For studies that lacked precise location data, we assigned coordinates based on locality descriptions in studies. We used the *spocc* R package to aggregate occurrence data [17].

**Table 1:** Tick species included in the modeling, and the diseases for which each is a competent pathogen vector, along with associated references

Tick species	Livestock diseases			
	Anaplasmosis	Babesiosis	Theileriosis	Heartwater
*Dermacentor albipictus* [18–22]	×			
*Dermacentor andersoni* [20–27]	×			
*Dermacentor hunteri* [28]	×			
*Dermacentor occidentalis* [20, 21, 24, 26, 29]	×			
*Dermacentor variabilis* [20–22, 25, 26, 30]	×			
*Ixodes scapularis* [20–22, 31]	×			
*Rhipicephalus annulatus* [20, 21, 32, 33]	×	×		
*Rhipicephalus microplus* [20, 21, 32, 33]	×	×		
*Rhipicephalus sanguineus* [20–22, 31, 34–36]	×			
*Haemaphysalis longicornis* [8, 37]			×	
*Amblyomma maculatum* [38–40]				×
*Amblyomma mixtum* [9, 38–40]				×
*Amblyomma variegatum* [9, 39–44]				×

Each tick species requires either one or three hosts to complete its life-cycle, with ungulates serving as key hosts in both life-cycle strategies [45]. Thus, we used occurrence data of native and exotic ungulates in North America as variables in tick models; data were compiled using GBIF, iNaturalist and the *spocc* R package. A total of 16 native and 15 exotic species formed the host database. As several of these species have overlapping distributions, we created distribution types, or chorotypes, for the 31 species. Chorotypes are particular distribution patterns that are significantly shared by a group of species, or the distribution pattern of a single species that does not overlap significantly with any other species [46]. Creating chorotypes prevents any species from being excluded from models due to distributions represented by other species, which ensures that comprehensive ungulate records are used. To identify chorotypes, we created a presence–absence matrix for the ungulate species that were then classified hierarchically using the Baroni-Urbani and Buser similarity index and the unweighted pair group method with an arithmetic mean agglomerative algorithm. Resulting clusters were assessed using the *RMacoqui* package in R [16, 46–48] with the internal homogeneity–distinctness index (IH), which measures the predominance of significant similarities within a candidate cluster versus between clusters. Clusters were accepted as chorotypes when IH = 1 or IH > 0 and was significant by a G-test. Single-species chorotypes arise when a species branches off distinctly from the rest.

### Predictor variable database

Along with the distribution of ungulate species and chorotypes, tick models incorporated 56 variables encompassing topo-hydrographic, anthropic, ecosystem and climatic factors (Additional file 1: Table S1). In addition to abiotic variables that may affect ticks, we considered spatial factors that may influence evolutionary and ecological processes that restrict or facilitate tick distribution, such as shifts. This spatial structure was incorporated using trend surface analysis, which generates a continuous surface representing broad-scale geographic patterns in species occurrence—i.e. the spatial trend—based on the coordinates of each location [49].

### Tick distribution models

Before creating models, we checked for multicollinearity among the explanatory variables using Spearman non-parametric correlation analysis. When two variables had a correlation higher than 0.8, the Rao’s score test was used to eliminate the least informative variable [50]. Due to the large number of variables, we control the Type I error using the false discovery rate (FDR) correction method [51]. Only variables with a significance level of *α* < 0.05 and an FDR-adjusted *q*-value < 0.05 were retained as significantly associated with species distribution. The variables retained were used in a multivariate stepwise logistic regression, a machine learning algorithm in which variables are added to a null model if they significantly improve the regression. Variable selection was based on the *P* value, employing ‘both’ direction strategy (forward and backward selection) to identify the most parsimonious and predictive model [52]. Probability values from regressions were then transformed into favorability values ranging from 0 to 1 using the favorability function [52]. Favorability models have been widely applied in modeling vectors and vector-borne diseases [53–57], as favorability values remove the effects of prevalence of each probability values and facilitate the comparison and integration of model outputs using fuzzy set theory tools [58]. These favorability values represent the favorable conditions predicted by the model at each hexagonal unit, which, at a broad scale, represents the potential distribution of the modeled species. Model development, and calculation of favorability values for each hexagonal unit, was performed using the *fuzzySim* R package [59].

We evaluated model classification accuracy and discrimination ability using the *modEvA* R package [60]. Sensitivity, specificity, underprediction, overprediction, kappa, correct classification rate and the true skill statistic were calculated, with prevalence used as the classification threshold [60, 61]. The model's discrimination ability was assessed with the area under the curve (AUC) from the receiver operating characteristic (ROC) curve, which measures a model's effectiveness distinguishing between outcomes across prediction thresholds. Once models of the potential distribution of ticks were developed using presence data (0 or 1), different grid cells scored in terms of favorability—i.e. the degree to which environmental and host-related conditions were suitable for each tick species, as indicated by the favorability values derived from the model. The number of records in each grid cell was used to validate the favorability values (0–1), assuming areas with more favorable conditions tend to have more tick records. For this purpose, favorability values above 0.5 were used to indicate the likely presence of ticks even in areas where they were not detected due to underreporting or limited sampling efforts. Based on the favorability function framework, a value of 0.5 represents the threshold between favorable and unfavorable environmental conditions because at this level probability is equal to the overall proportion of areas with ticks [52, 62, 63]. The outputs of the different tick species models were used to assess relationships between tick diversity and ungulate diversity. We tested whether areas with higher potential to host more tick species also coincided with areas hosting a greater number of ungulate species, providing insight into how host community structure may influence the spatial patterns of tick-borne pathogen risk.

### Modeling disease risk for cattle

**Fig. 1:**
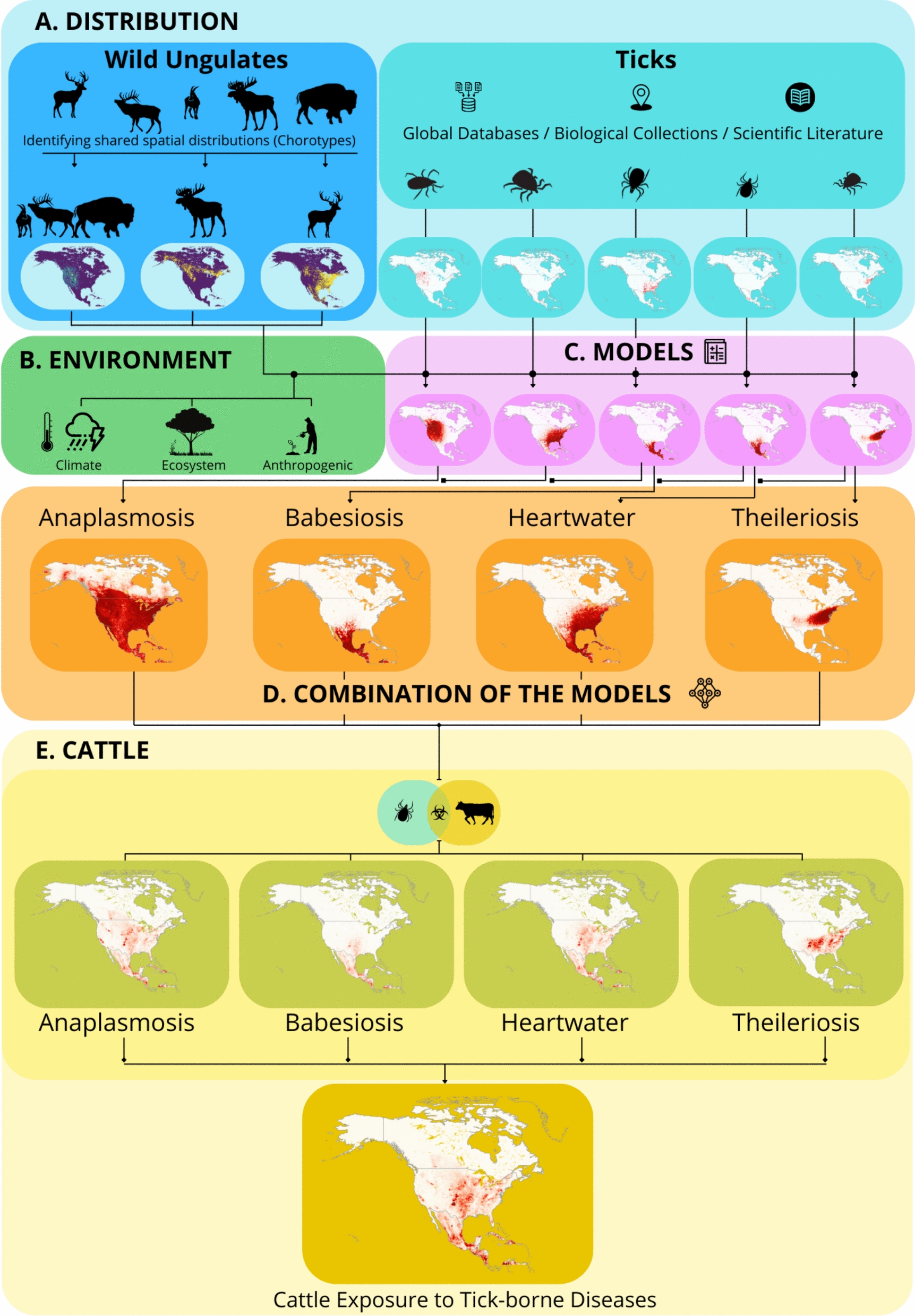
Approach for model development. We leveraged tick and ungulate occurrence records (**A**) and environmental variables (**B**) to create distribution models for 13 tick species (**C**). **D** Tick distribution models were combined based on their competence in transmitting each of the pathogens that cause four major livestock diseases (anaplasmosis, babesiosis, heartwater, theileriosis). **E** Models were intersected with a cattle distribution density to map the risk of each disease. Maps showing potential exposure to different diseases were then merged into a single map which highlights favorability of cattle-tick interactions across North America

Once we developed the distribution models for the 13 tick species, we combined specific tick models based on their capacity to transmit specific pathogens (Fig. 1). As a single competent species in an area is all that is needed to transmit a pathogen, we integrated models of competent vectors using a fuzzy union approach from fuzzy logic theory, implemented here by selecting the maximum favorability value (F) across all vector species in each grid cell (i.e. F-vector_1_, ⋃ F-vector_2_ ,  ⋃ F-vector_n_). With this approach, high-risk areas were identified as those with highly favorable conditions for at least one competent tick species, irrespective of favorability levels for other competent tick species. Next, to estimate the risk of cattle exposure to each pathogen, we performed a fuzzy intersection between results of the fuzzy union for each tick group and the normalized spatial distribution of cattle density (scaled between 0 and 1 using min–max normalization) for 2020 [64, 65]. This ensures comparability with the favorability values, which are also bounded between 0 and 1. We selected the minimum values of favorability and normalized cattle density in North America for each competent tick species (i.e., F_ticks_ ∩ Density_cattle_), allowing for the spatial identification of areas with the highest risk for cattle exposure. Regions with high cattle density and high favorability for vector ticks are classified as high-risk zones for exposure. Conversely, areas with low cattle density and/or low favorability for vector ticks are classified as low-risk zones for cattle exposure.

Overall, our modeling approach generated four risk maps of cattle exposure to ticks, one for each disease: (i) anaplasmosis, (ii) babesiosis, (iii) heartwater and (iv) theileriosis. Each map showed a risk value within each geographic unit. We also mapped the risk of cattle exposure to all tick-borne diseases across North America, where the overall risk in each geographic unit was calculated as the cardinality of the diseases for which cattle are at risk. Consequently, areas with the highest overall risk are those with elevated risk for multiple tick-borne pathogens (Fig. 1). Finally, to quantify contribution of ungulate hosts to the risk maps, we developed additional risk models that did not include any of the wildlife hosts as input variables, and then calculated the overlap between the risk maps including ungulates and excluding them [59]. This allowed us to determine how maps with or without ungulates performed in spatial validations.

## Results

### Tick vector and ungulate host databases

**Fig. 2:**
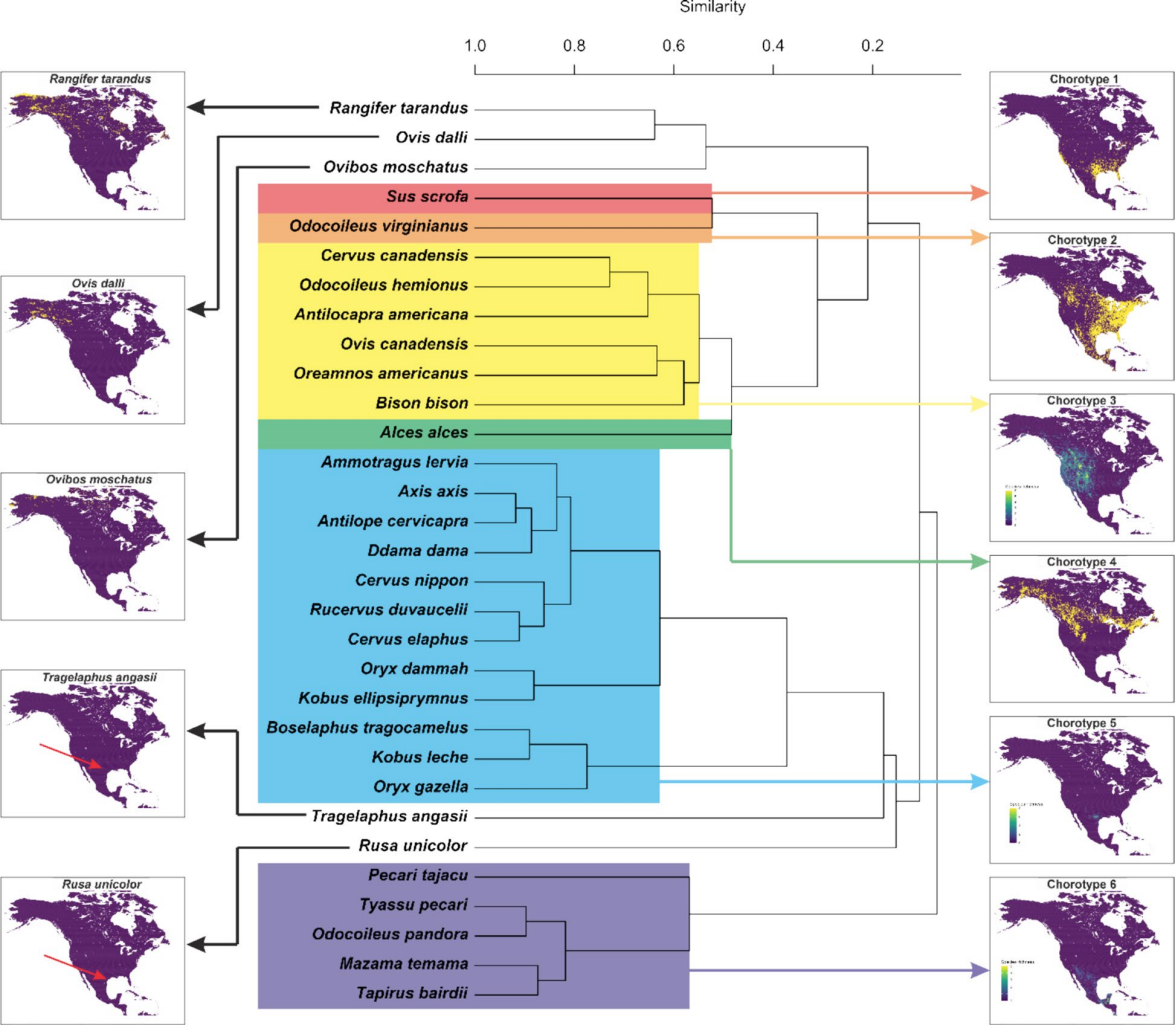
Chorotype analysis to classify the 31 ungulate species in North America based on their distributional similarities (Baroni-Urbani and Buser similarity index). Species in each colored box form a chorotype, while species outside boxes exhibit a gradual spatial distribution pattern

We compiled 102,665 records for the 13 tick species known to transmit pathogens causing the four livestock diseases in North America, Central America and the Caribbean. These ranged from the species with the most records, *Dermacentor variabilis* (39,548), to *A. variegatum*, which is primarily restricted to the Caribbean (25). We compiled 412,333 records across 16 native and 15 exotic ungulate hosts, encompassing widely distributed species such as white-tailed deer (*Odocoileus virginianus*) (118,733 records) and more localized exotic species introduced on game ranches, such as nilgai (*Boselaphus tragocamelus*) (428). Chorotype analysis revealed the presence of six significantly different biogeographical distribution patterns (chorotypes) among the ungulate species, grouping 26 species in the different chorotypes. In contrast, five individual species did not constitute any chorotype and followed a more gradual spatial pattern (Fig. 2).

### Distribution modeling of tick species

Models for the 13 tick species (Additional file 1: Figure S1) had high sensitivity (mean 0.95, standard error [SE] 0.014) and specificity (mean 0.88, SE 0.024), and the true skill statistic of all models indicated a good balance between sensitivity and specificity (mean 0.84, SE 0.036) (Additional file 1: Tables S2, S3). All models had high AUCs (mean 0.96, SE 0.013), indicating they effectively discriminated tick distributions (Additional file 1: Table S2). Model underprediction was very low (mean 0.0014, SE 0.00067), meaning critical omissions that could compromise cattle health were minimized (Additional file 1: Table S2). Moreover, hexagonal units with higher favorability values had a greater number of tick records (Additional file 1: Figure S2). For most of the species, the classification and discrimination capacity of models increased when ungulate hosts were included as an explanatory variable (Additional file 1: Figure S3).

### Modeling risk of tick-borne pathogens

**Fig. 3:**
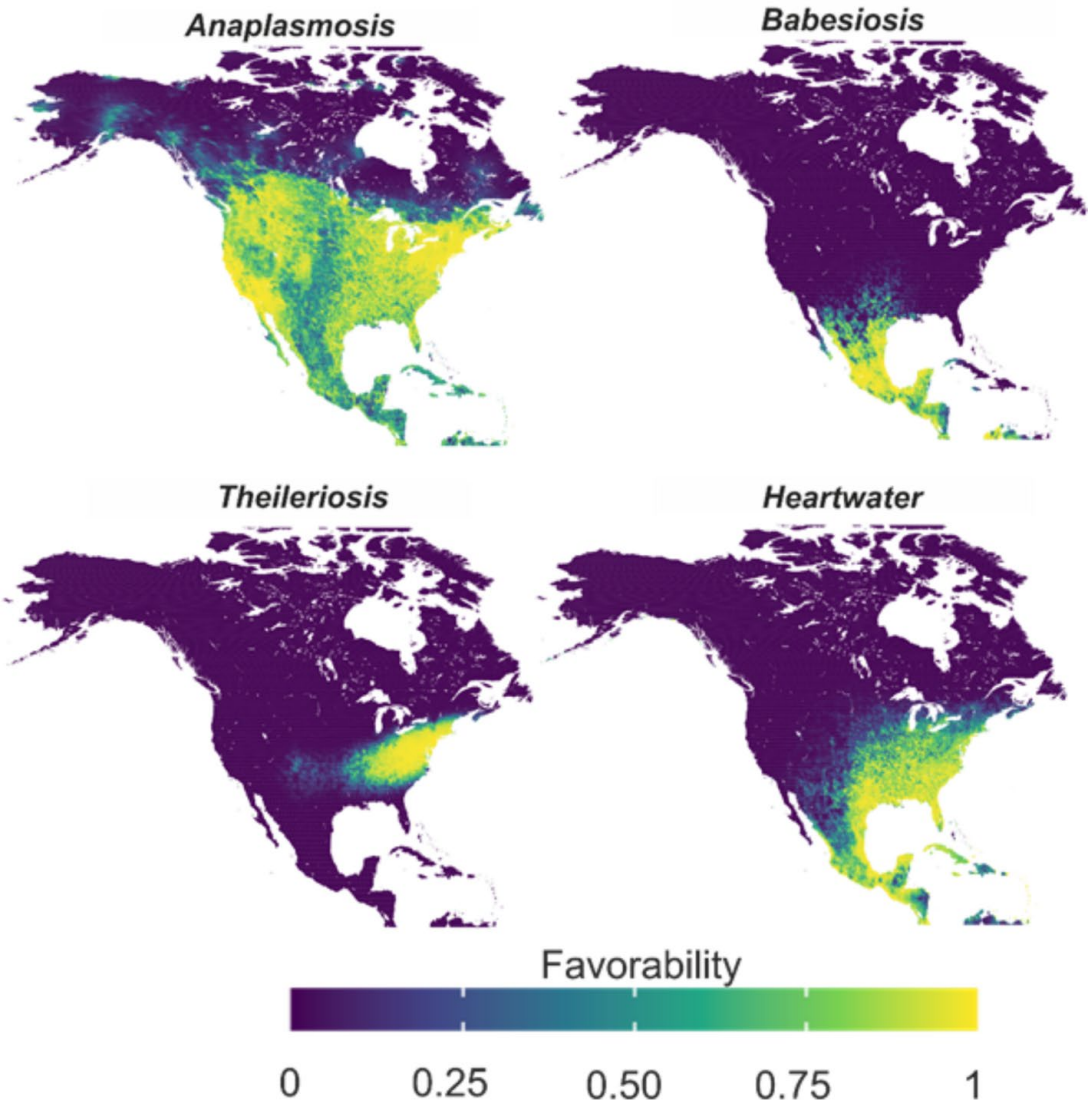
Combination of the 13 tick species models, assessing their capacity to transmit the pathogens associated with anaplasmosis, babesiosis, heartwater and theileriosis diseases

The exposure model for anaplasmosis showed the widest potential distribution, with favorable conditions for tick vectors and cattle across the entirety of Central America, Mexico, the USA and southern Canada (Fig. 3). Babesiosis, in contrast, was limited to Mexico and Texas (Fig. 3). Heartwater had favorable habitats throughout Mexico and the entire eastern USA, whereas theileriosis was limited to the Mid-Atlantic, Midwest and northeastern USA (Fig. 3). By combining risk models, we found that the overall risk of cattle exposure to all tick-borne diseases is most prominent in southern Mexico (Additional file 1: Figure S4). In the USA, the center of the country has the highest risk of exposure to tick-borne diseases, with the eastern half of the country showing far greater risk than the western (Additional file 1: Figure S4). In the Caribbean, risk is spread across all the islands. In Canada, only the southern part faces major risk, including the interior plains and the Great Lakes region (Additional file 1: Figure S4).

**Fig. 4:**
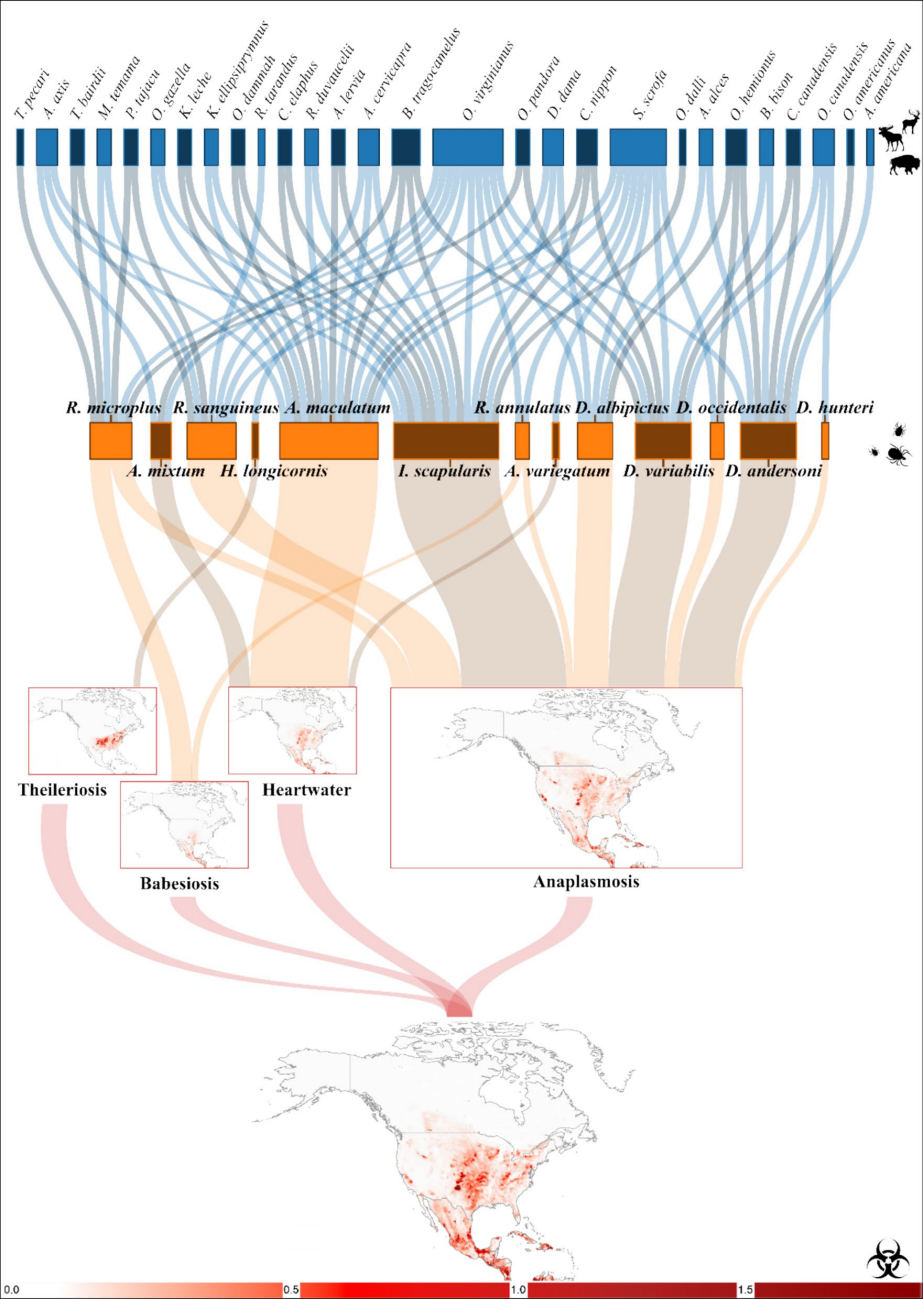
Relationships between wild ungulates, tick species and tick-borne disease exposure in North America. Flows connect ungulates (blue boxes) to the distribution of tick species (orange boxes), as well as to ticks associated with the pathogens they transmit. The size of the blue boxes indicates the number of tick species to which ungulates contribute, and the size of the orange boxes indicates the number of ungulate species involved in the distribution of each tick. Transition from light to dark red indicates increasing level of tick-borne disease exposure risk to cattle

Nine tick species have the potential to transmit the bacterium that causes anaplasmosis, and 28 of the 31 ungulate species in North America were significantly associated with these ticks (Fig. 4). These nine tick species are distributed across much of the Americas, from Canada to the Caribbean, with the most favorable habitat along the eastern and western coasts of the USA. Cattle at the highest risk of anaplasmosis exposure are concentrated in the Midwest and southern USA, as well as in the Caribbean region and along Mexico's Caribbean coast (Fig. 4). Similarly, the three *Amblyomma* species associated with heartwater were associated with 14 ungulate species (Fig. 4). These ticks have a favorable habitat in the Caribbean islands and parts of the USA and Mexico bordering the Gulf of Mexico. These regions are likely to have the greatest risk of heartwater exposure if the pathogen spreads (Fig. 4).

The two cattle fever tick species involved in the transmission of babesiosis (*Rhipicephalus microplus* and *Rhipicephalus annulatus*) were only significantly associated with seven ungulate species (Fig. 4), and a highly favorable habitat was concentrated in southern Mexico. However, favorable conditions for these vectors were also present in northern Mexico and Texas (Fig. 4). Finally, only the exotic *H. longicornis* can transmit the Ikeda genotype of *T. orientalis*. The widely distributed white-tailed deer is significantly associated with the distribution of this tick (Fig. 4). The southern USA is most favorable for *H. longicornis*, placing cattle in Texas and neighboring states at the highest risk of exposure to theileriosis (Fig. 4).

Overall, the white-tailed deer was by far the ungulate species most strongly associated with the distribution of ticks affecting cattle, being significantly linked to the distribution of 10 out of the 13 tick species. White-tailed deer were also significantly associated with the distribution of each of the four diseases studied. Ungulates, such as the exotic wild boar (*Sus scrofa*), were also broadly associated with several tick species. Among tick species, *Ixodes scapularis* and *Amblyomma maculatum* were linked to most ungulate species, with associations to 15 and 14 species, respectively (Fig. 4). Overall, our analyses revealed a significant and positive relationship between tick diversity and ungulate diversity (Additional file 1: Figure S5). The overlap between the risk model including ungulate hosts and the one excluding them was 97%, suggesting that 3% of the risk distribution was attributable to the contribution of ungulates (Additional file 1: Figure S6).

## Discussion

Our study mapped the potential risk of cattle exposure to tick-borne pathogens across North America by assessing intricate relationships between ticks, ungulate hosts and environmental conditions. Our species distribution models improved when incorporating ungulate hosts, enabling us to identify the key ungulate species shaping tick distributions.

Bovine anaplasmosis poses the most widespread tick-borne disease risk in North America, including the Caribbean, Mexico, the USA and southern Canada. Its broad distribution is linked to multiple tick vectors and a diverse range of wild ungulate hosts. Although eradicated from Canadian livestock, the disease remains a threat due to persistent vectors and wildlife reservoirs [66, 67]. Our models identified 28 ungulate species as potential hosts, underscoring their role in tick maintenance and disease spread. These results highlight the need for better diagnostics and surveillance targeting wild ruminants, both being crucial for managing a disease that costs the USA an estimated $300 million annually [36, 68].

The risk of babesiosis is widespread across Mexico due to the complementary distributions of *R. microplus* and *R. annulatus*, two tick species suited to tropical and temperate zones, respectively. Their overlap in southern Mexico leads to higher exposure and reported disease prevalence of up to 90%, compared to 50% in the north [69, 70]. Models show a strong association between both of these ticks and white-tailed deer, a key secondary host in Mexico [71] and the main vector introducer into the USA. [72, 73]. Managing the expansion of these ticks and their hosts is essential to reduce the economic burden of babesiosis in Mexico, estimated at US$573.6 million annually [74], and to prevent its spread into the USA.

*Haemaphysalis longicornis*, the vector of theileriosis, was recently introduced into the USA.; however, it shows high predicted favorability along the East Coast. Models also highlight favorable areas in states where the tick has not yet been reported. At a broader scale, its distribution is strongly associated with white-tailed deer, consistent with US Department of Agriculture reports and other studies [75–77]. This relationship may facilitate its westward expansion, with models already predicting suitable habitats inland and into northern Mexico.

The Caribbean, where heartwater and its vector *A. variegatum* are present, represents the highest-risk area for livestock. However, our models identify high-risk areas on the mainland as well, due to the presence of other competent vectors, such as *A. maculatum* and *Amblyomma mixtum*. Wild boars, which are associated with heartwater vectors in both the Caribbean and mainland regions, may further facilitate the spread [78–80]. If the pathogen reaches the mainland, whether through animal trade or due to the natural dispersal of hosts such as Cattle Egrets [9, 81, 82], it could become established through alternative vectors and suitable host populations. Early detection is critical, as experience in the Caribbean shows that once established, heartwater is extremely difficult to eradicate.

Although the One Health approach, which recognizes the interconnectedness of humans, animals and environment, is often advocated for tick-borne pathogens [14], tick and tick-borne pathogen models typically account for environmental factors but neglect the host dimension [3, 13, 83]. This has created a knowledge gap limiting our understanding of risk of tick-borne pathogens, especially given that potential importance of considering host populations to model the risk of ticks and tick-borne disease is well-documented [84–86]. This omission is common not only in tick-borne diseases but also in vector-borne diseases more broadly [87].

Although our disease risk models that included hosts showed only a 3% mean difference compared to models that did not, this likely underestimates the effects of hosts. Incorporating hosts into tick models improved their accuracy and provided insights into the relationships between specific ungulate species, tick species and associated pathogens, which could enable more targeted monitoring, as ungulates act as bridges between domestic livestock and natural ecosystems [88]. In addition to studies linking ungulate abundance and diversity to tick abundance [89, 90], our study suggests that higher ungulate diversity supports a greater variety of tick species across North America. While host diversity may reduce disease risk via a ‘dilution effect’ [11] and although many of these ungulates are not known reservoirs for many livestock pathogens, these hosts can still provide blood meals to increase vector abundance and diversity, potentially raising the risk of tick-borne disease [91, 92]. While relatively understudied, the net effect of host diversity on tick-borne pathogen risk depends on interactions among many wildlife host and tick vector species.

Our use of chorotypes to characterize host communities offers a valuable approach for analyzing multiple species simultaneously, allowing for the assessment of host distribution in relation to the geographical patterns of pathogen risk [16, 48, 53]. Integrating risk models for multiple diseases offers a more comprehensive understanding of epidemiological dynamics, enabling the identification of high-risk areas that require focused attention. Rather than simply targeting areas with a high risk of a single disease, this integrated approach highlights regions where livestock may be simultaneously exposed to multiple pathogens, providing a more holistic view of risk. This integrated risk map can also serve as decision-support tools, guiding surveillance and prevention efforts by helping prioritize areas where targeted interventions may be most needed.

While our approach offers valuable insights, it has limitations. Model accuracy depends on the quality of occurrence data, which may be spatially biased or incomplete, particularly in under-sampled regions. Although we included environmental, vector, host and cattle data, broad-scale information on pathogen prevalence is lacking and would improve risk estimates. Given the complexity of host–vector–pathogen interactions, our models offer a first approximation to guide surveillance. Integrating pathogen detection or prevalence data from ticks and hosts in future studies will help refine predictions and enhance our ecological understanding of tick-borne diseases.

## Conclusions

This study provides a comprehensive approach to understanding the large-scale risk of tick-borne pathogens affecting cattle in North America, with an emphasis on the need to incorporate environmental and host factors into models. Our findings highlight the key role of wild ungulates, showing a positive correlation with both vector diversity and improved tick distribution predictions. Continuous monitoring and the availability of empirical data are essential for validating models and ensuring their accuracy and reliability. Our approach provides a clear understanding of transmission dynamics and highlights the need to implement monitoring and control strategies that consider both the environmental context and host populations. Moving forward, it is essential to continue assessing how interactions between hosts, vector diversity, and specific pathogen species may affect disease prevalence. Through an integrated One Health approach that leverages vector, host, and abiotic data, risks associated with vector-borne pathogens can be more effectively mitigated.

## Supplementary Information

**Additional file1: Table S1.** Explanatory variables used in the tick models.** Table S2.** Evaluation of the tick models.** Table S3.** Mathematical models for the different tick species.** Figure S1. **Distributional models for the different tick species.** Figure S2. **Favorability values of the models *vs* number of tick records at each grid cell.** Figure S3. **Tick model performance with and without the inclusion of hosts (ungulates).** Figure S4.** Predicted risk of exposure of North American cattle to the different tick-borne diseases as well as the combination of all four diseases.** Figure S5.** Relationship between ungulate species diversity and tick species diversity.** Figure S6.** Risk of exposure of North American cattle to tick-borne diseases considering hosts and excluding them.

## Data Availability

The datasets supporting this study are available as follows: cattle data from the FAO (https://www.fao.org/livestock-systems/global-distributions/cattle/en); environmental data in the Supplementary Information; and vector and host datasets on Figshare at the following link: [https://figshare.com/s/2ef90547c97e1c6dbf99].

## Abbreviations

AUC: Area under the curve
F: Favorability
FDR: False discovery rate
GBIF: Global Biodiversity Information Facility
IH: Internal homogeneity
ROC: Receiver operating characteristic
