# Supplementary material for: Linking tick and wildlife host distributions to map risk of tick-borne diseases
Source: Parasit Vectors. 2025 Nov 19;18:472. doi: 10.1186/s13071-025-07096-0 (PMC12628546; doi:10.1186/s13071-025-07096-0)

**Supporting Information for:**

Linking Tick and Wildlife Host Distributions to Map Risk of Tick-Borne Diseases

**Table S1.** Explanatory variables in the tick models and associated references.

| **Factor** | **Variable** | **Variable Name and Reference** |
| --- | --- | --- |
| Topo-hydrographic | alt | Altitude^1^ |
|  | slope | Slope^2^ |
|  | dist_river | Distance to rivers^3^ |
| Anthropogenic | pop_dens | Human concentration^4^ |
|  | dist_pop | Distance to population center^5^ |
|  | dist_rail | Distance to roads^6^ |
|  | dist_road | Distance to railway tracks^6^ |
|  | class11 | Post-flooding or irrigated croplands^7^ |
|  | class14 | Rainfed croplands^7^ |
|  | class20 | Mosaic cropland (50-70%) / vegetation (grassland, shrubland, forest) (20-50%)^7^ |
|  | class30 | Mosaic vegetation (grassland, shrubland, forest) (50-70%) / cropland (20-50%)^7^ |
| Ecosystem | class40 | Closed to open (>15%) broadleaved evergreen and/or semi-deciduous forest (>5m) ^7^ |
|  | class50 | Closed (>40%) broadleaved deciduous forest (>5m)^7^ |
|  | class60 | Open (15-40%) broadleaved deciduous forest (>5m)^7^ |
|  | class70 | Closed (>40%) needleleaved evergreen forest (>5m)^7^ |
|  | class90 | Open (15-40%) needleleaved deciduous/evergreen forest (>5m)^7^ |
|  | class100 | Closed to open (>15%) mixed broadleaved and needleleaved forest (>5m)^7^ |
|  | class110 | Mosaic forest/shrubland (50-70%) / grassland (20-50%)^7^ |
|  | class120 | Mosaic grassland (50-70%) / forest / shrubland (20-50%)^7^ |
|  | class130 | Closed to open (>15%) shrubland (<5m)^7^ |
|  | class140 | Closed to open (>15%) grassland^7^ |
|  | class150 | Sparse (>15%) vegetation (woody vegetation, shrubs, grassland)^7^ |
|  | class160 | Closed (>40%) broadleaved forest regularly flooded - fresh water^7^ |
|  | class170 | Closed (>40%) broadleaved semi-deciduous and/or evergreen forest regularly flooded - saline water^7^ |
|  | class180 | Closed to open (>15%) vegetation (grassland, shrubland, woody vegetation) on regularly flooded or waterlogged soil - fresh, brackish or saline water^7^ |
|  | class190 | Artificial surfaces and associated areas (urban areas >50%)^7^ |
|  | class200 | Bare areas^7^ |
|  | class210 | Water bodies^7^ |
|  | class220 | Permanent snow and ice^7^ |
|  | ndvi_mean | Mean value of the NDVI from 1999 to 2019^8^ |
|  | ndvi_min | Minimum value of the NDVI from 1999 to 2019^8^ |
|  | ndvi_max | Maximum value of the NDVI from 1999 to 2019^8^ |
|  | ndvi_diff | Range between the maximum and minimum value of the NDVI from 1999 to 2019^8^ |

**Table S1 (continued).** Explanatory variables in the tick models and associated references.

| **Factor** | **Variable** | **Variable Name and Reference** |
| --- | --- | --- |
| Climatic | bio1 | mean annual air temperature^9^ |
|  | bio2 | mean diurnal air temperature range^9^ |
|  | bio3 | isothermality^9^ |
|  | bio4 | temperature seasonality^9^ |
|  | bio5 | mean daily maximum air temperature of the warmest month^9^ |
|  | bio6 | mean daily minimum air temperature of the coldest month^9^ |
|  | bio7 | annual range of air temperature^9^ |
|  | bio8 | mean daily mean air temperatures of the wettest quarter^9^ |
|  | bio9 | mean daily mean air temperatures of the driest quarter^9^ |
|  | bio10 | mean daily mean air temperatures of the warmest quarter^9^ |
|  | bio11 | mean daily mean air temperatures of the coldest quarter^9^ |
|  | bio12 | annual precipitation amount^9^ |
|  | bio13 | precipitation amount of the wettest month^9^ |
|  | bio14 | precipitation amount of the driest month^9^ |
|  | bio15 | precipitation seasonality^9^ |
|  | bio16 | mean monthly precipitation amount of the wettest quarter^9^ |
|  | bio17 | mean monthly precipitation amount of the driest quarter^9^ |
|  | bio18 | mean monthly precipitation amount of the warmest quarter^9^ |
|  | bio19 | mean monthly precipitation amount of the coldest quarter^9^ |
|  | hurs_mean | Mean monthly near-surface relative humidity^9^ |
|  | hurs_max | Maximum monthly near-surface relative humidity^9^ |
|  | hurs_min | Minimum monthly near-surface relative humidity^9^ |
|  | hurs_range | Annual range of monthly near-surface relative humidity^9^ |

**References for Table S1**

^1^ GTOPO30 (US Geological Survey 1996).

^2^ Elaborated from Digital Elevation Model using the altitude variable GTOPO30, US Geological Survey 1996, using the Geographic Information Sistem ArcGIS Desktop 10.3.

^3^ Global Drainage Basin Database. http://www.cger.nies.go.jp/db/gdbd/gdbd_index_e.html.

^4^ Landscan 2000 Global Population Database. https://landscan.ornl.gov.

^5^ Administrative Centers & Populated Places shapefile at the Relational World Database II (RWDB2) updated in 2000. http://www.fao.org/geonetwork.

^6^ Vector Map Level 0 at the Digital Chart of the World. http://worldmap.harvard.edu.

^7^ GLOBCOVER. https://due.esrin.esa.int/page_globcover.php.

^8^ Copernicus Land Monitoring Service. https://land.copernicus.eu.

^9^ CHELSA. http://chelsa-climate.org

**Table S2.** Comparative assessment of the classification and discrimination capacities of the different tick models in North America. AUC: area under the receiver operating characteristic curve.

| **Tick Species** | **Sensitivity** | **Specificity** | **Correct Classification**  **Rate** | **Kappa** | **True Skill Statistic** | **Under-prediction** | **Over-prediction** | **AUC** |
| --- | --- | --- | --- | --- | --- | --- | --- | --- |
| *D. albipictus* | 0.82 | 0.68 | 0.69 | 0.016 | 0.51 | 1.3E-03 | 0.99 | 0.83 |
| *D. andersoni* | 0.96 | 0.86 | 0.86 | 0.13 | 0.82 | 6.0E-04 | 0.92 | 0.96 |
| *D. hunteri* | 0.99 | 0.96 | 0.96 | 0.098 | 0.95 | 3.0E-05 | 0.95 | 0.99 |
| *D. occidentalis* | 0.99 | 0.98 | 0.98 | 0.41 | 0.97 | 5.8E-05 | 0.74 | 1.0 |
| *D. variabilis* | 0.94 | 0.83 | 0.84 | 0.47 | 0.77 | 8.8E-03 | 0.61 | 0.94 |
| *I. scapularis* | 0.96 | 0.89 | 0.90 | 0.50 | 0.85 | 3.2E-03 | 0.62 | 0.97 |
| *R. annulatus* | 0.96 | 0.94 | 0.94 | 0.090 | 0.90 | 1.5E-04 | 0.95 | 0.99 |
| *R. microplus* | 0.97 | 0.93 | 0.93 | 0.091 | 0.89 | 1.5E-04 | 0.95 | 0.99 |
| *R. sanguineus* | 0.89 | 0.76 | 0.76 | 0.069 | 0.65 | 2.0E-03 | 0.95 | 0.91 |
| *H. longicornis* | 0.97 | 0.95 | 0.95 | 0.23 | 0.92 | 2.4E-04 | 0.87 | 0.99 |
| *A. maculatum* | 0.96 | 0.89 | 0.89 | 0.28 | 0.85 | 1.2E-03 | 0.82 | 0.97 |
| *A. mixtum* | 0.99 | 0.92 | 0.92 | 0.088 | 0.90 | 6.2E-05 | 0.95 | 0.98 |
| *A. variegatum* | 1.00 | 0.90 | 0.91 | 0.37 | 0.90 | 0.00 | 0.75 | 0.98 |
| Average | 0.95 | 0.88 | 0.89 | 0.22 | 0.84 | 1.3E-03 | 0.85 | 0.96 |

**Table S3.** Variables included in the different tick models. *Sptrend* refers to the spatial structure. *Estimate* is the coefficient that multiplies the variable values in the logit of the multivariate logistic regression. The *Wald* parameter quantifies the relevance of the variable in the model.

|  | **Dermacentor albipictus** | | **Dermacentor andersoni** | | **Dermacentor hunteri** | |
| --- | --- | --- | --- | --- | --- | --- |
| **Variable** | **Estimate** | **Wald** | **Estimate** | **Wald** | **Estimate** | **Wald** |
| **dist_rail** | -9.58E-06 | 1.24E+01 | -6.45E-06 | 4.13E+00 | - | - |
| **dist_pop** | - | - | -4.95E-06 | 3.27E+00 | - | - |
| **dist_road** | - | - | -7.31E-05 | 1.06E+01 | - | - |
| **bio18** | -1.57E-04 | 4.25E+00 | -4.63E-04 | 2.03E+01 | - | - |
| **bio5** | - | - | -1.09E-02 | 1.86E+01 | - | - |
| **bio2** | - | - | 1.25E-02 | 4.35E+00 | - | - |
| **hurs_mean** | - | - | - | - | -1.79E-03 | 1.18E+01 |
| **class70** | 1.17E+00 | 1.05E+01 | - | - | - | - |
| **class90** | -2.32E+00 | 6.38E+00 | - | - | - | - |
| **class30** | - | - | 2.55E+00 | 1.87E+01 | - | - |
| **class100** | - | - | -3.57E+00 | 1.50E+01 | - | - |
| **class140** | - | - | -1.63E+00 | 8.61E+00 | - | - |
| **class120** | - | - | - | - | 1.06E+01 | 1.17E+01 |
| **ndvi_diff** | - | - | - | - | -6.79E+00 | 6.46E+00 |
| **alt** | - | - | -5.29E-04 | 1.31E+01 | -1.60E-03 | 2.59E+01 |
| **slope** | - | - | 7.86E-02 | 7.63E+00 | 4.49E-01 | 3.20E+01 |
| **chor_2** | 8.84E-01 | 2.56E+01 | 3.83E-01 | 8.02E+00 | - | - |
| **O.hemionus** | 5.37E-01 | 6.74E+00 | - | - | - | - |
| **chor_4** | 9.18E-01 | 2.53E+01 | 4.22E-01 | 1.08E+01 | - | - |
| **D.dama** | 1.27E+00 | 6.84E+00 | - | - | - | - |
| **chor_1** | 4.54E-01 | 4.12E+00 | - | - | - | - |
| **chor_3** | - | - | 3.37E-01 | 5.60E+01 | - | - |
| **O.canadensis** | - | - | - | - | 7.64E-01 | 6.68E+00 |
| **sptrend** | - | - | 6.76E-01 | 1.34E+02 | 5.41E-15 | 1.13E+01 |
| **(Intercept)** | -5.15E+00 | 4.24E+02 | 3.17E+01 | 1.85E+01 | 8.71E+00 | 1.92E+01 |

**Table S3 (continued).** Variables included in the different tick models. *Sptrend* refers to the spatial structure. *Estimate* is the coefficient that multiplies the variable values in the logit of the multivariate logistic regression. The *Wald* parameter quantifies the relevance of the variable in the model.

|  | **Dermacentor occidentalis** | | **Dermacentor variabilis** | | **Ixodes scapularis** | |
| --- | --- | --- | --- | --- | --- | --- |
| **Variable** | **Estimate** | **Wald** | **Estimate** | **Wald** | **Estimate** | **Wald** |
| **dist_pop** | -3.03E-05 | 1.65E+01 | -1.38E-05 | 7.32E+01 | -1.35E-05 | 3.27E+01 |
| **dist_road** | -2.24E-04 | 1.58E+01 | -4.49E-05 | 2.09E+01 | -9.04E-05 | 3.48E+01 |
| **dist_rail** | -9.01E-06 | 3.84E+00 | -2.78E-05 | 1.22E+02 | -2.96E-05 | 4.89E+01 |
| **bio15** | 3.79E-03 | 9.49E+00 | - | - | 1.11E-03 | 6.44E+00 |
| **bio2** | 3.68E-02 | 2.27E+01 | -1.34E-02 | 4.01E+01 | - | - |
| **hurs_range** | -1.03E-03 | 8.34E+00 | - | - | - | - |
| **hurs_max** | 1.11E-03 | 6.60E+00 | -7.39E-04 | 7.15E+01 | - | - |
| **bio8** | - | - | - | - | 1.65E-03 | 7.25E+00 |
| **bio14** | - | - | -5.80E-04 | 1.79E+01 | -1.04E-03 | 1.03E+01 |
| **hurs_min** | - | - | - | - | 6.46E-04 | 7.54E+00 |
| **bio4** | - | - | 2.99E-04 | 1.85E+02 | - | - |
| **bio18** | - | - | -1.83E-04 | 3.39E+01 | - | - |
| **class70** | 1.84E+00 | 1.13E+01 | 1.63E+00 | 5.87E+01 | 1.30E+00 | 1.62E+01 |
| **class50** | - | - | 1.47E+00 | 6.63E+01 | 1.46E+00 | 3.87E+01 |
| **class160** | - | - | 4.11E+01 | 3.40E+01 | 4.26E+01 | 2.18E+01 |
| **class30** | - | - | 1.38E+00 | 1.98E+01 | 1.57E+00 | 1.58E+01 |
| **class220** | - | - | 3.63E+00 | 3.58E+00 | 1.57E+01 | 1.23E+01 |
| **class190** | - | - | 9.22E+00 | 6.77E+01 | 5.00E+00 | 9.95E+00 |
| **class90** | - | - | -2.88E+00 | 2.70E+01 | - | - |
| **class130** | - | - | -1.55E+00 | 2.03E+01 | - | - |
| **class120** | - | - | -4.37E+00 | 1.56E+01 | - | - |
| **class60** | - | - | 2.61E+01 | 4.53E+01 | - | - |
| **class40** | - | - | -1.18E+01 | 1.12E+01 | - | - |
| **class170** | - | - | 1.87E+00 | 1.26E+01 | - | - |
| **ndvi_diff** | -5.77E+00 | 1.13E+01 | 6.23E+00 | 1.18E+02 | -6.38E+00 | 5.67E+01 |
| **ndvi_min** | - | - | 1.10E+01 | 9.75E+01 | -1.52E+01 | 1.21E+02 |
| **ndvi_mean** | - | - | -8.74E+00 | 8.21E+01 | 1.15E+01 | 8.46E+01 |
| **slope** | 2.99E-01 | 1.87E+01 | 2.14E-01 | 6.88E+01 | 1.71E-01 | 8.72E+00 |
| **alt** | - | - | -1.21E-03 | 1.19E+02 | -1.46E-03 | 3.00E+01 |
| **dist_river** | - | - | -3.87E-06 | 4.76E+00 | 1.04E-05 | 2.81E+01 |

**Table S3 (continued).** Variables included in the different tick models. *Sptrend* refers to the spatial structure. *Estimate* is the coefficient that multiplies the variable values in the logit of the multivariate logistic regression. The *Wald* parameter quantifies the relevance of the variable in the model.

|  | **Dermacentor occidentalis** | | **Dermacentor variabilis** | | **Ixodes scapularis** | |
| --- | --- | --- | --- | --- | --- | --- |
| **Variable** | **Estimate** | **Wald** | **Estimate** | **Wald** | **Estimate** | **Wald** |
| **O.hemionus** | 1.87E+00 | 1.91E+01 | - | - | - | - |
| **chor_1** | 8.61E-01 | 1.08E+01 | 4.84E-01 | 4.55E+01 | 1.13E+00 | 1.34E+02 |
| **chor_2** | - | - | 1.25E+00 | 5.48E+02 | 1.16E+00 | 2.00E+02 |
| **chor_5** | - | - | - | - | 5.11E-01 | 3.47E+01 |
| **O.dalli** | - | - | - | - | 4.51E+00 | 8.97E+00 |
| **C.canadensis** | - | - | 3.81E-01 | 1.87E+01 | - | - |
| **O.pandora** | - | - | 5.91E+00 | 1.09E+01 | - | - |
| **B.bison** | - | - | 4.94E-01 | 1.34E+01 | - | - |
| **O.canadensis** | - | - | 3.98E-01 | 6.51E+00 | - | - |
| **C.nippon** | - | - | 8.53E-01 | 5.76E+00 | - | - |
| **B.tragocamelus** | - | - | 9.46E-01 | 4.96E+00 | - | - |
| **sptrend** | 5.06E-01 | 3.56E+01 | - | - | 8.62E-01 | 4.55E+02 |
| **(Intercept)** | -1.29E+01 | 1.73E+01 | 1.39E+00 | 3.96E+00 | -8.02E+00 | 1.06E+01 |

**Table S3 (continued).** Variables included in the different tick models. *Sptrend* refers to the spatial structure. *Estimate* is the coefficient that multiplies the variable values in the logit of the multivariate logistic regression. The *Wald* parameter quantifies the relevance of the variable in the model.

|  | **Rhipicephalus annulatus** | | **Rhipicephalus microplus** | | **Rhipicephalus sanguineus** | |
| --- | --- | --- | --- | --- | --- | --- |
| **Variable** | **Estimate** | **Wald** | **Estimate** | **Wald** | **Estimate** | **Wald** |
| **dist_rail** | -7.66E-06 | 5.58E+00 | -7.41E-06 | 9.77E+00 | -4.52E-03 | 2.87E+01 |
| **dist_pop** | -8.65E-05 | 3.07E+01 | -3.37E-05 | 1.00E+01 | -4.70E-05 | 1.12E+02 |
| **dist_road** | - | - | - | - | -1.50E-05 | 3.23E+00 |
| **bio8** | 9.20E-03 | 9.40E+00 | 4.08E-02 | 2.89E+01 | -4.52E-03 | 2.87E+01 |
| **bio2** | 1.03E-02 | 4.65E+00 | - | - | - | - |
| **bio15** | - | - | 1.51E-03 | 1.03E+01 | - | - |
| **hurs_max** | - | - | 7.34E-04 | 4.39E+00 | 4.29E-04 | 7.89E+00 |
| **bio1** | - | - | -2.62E-02 | 1.14E+01 | - | - |
| **class190** | 1.26E+01 | 3.25E+01 | - | - | 7.40E+00 | 9.71E+01 |
| **class30** | 2.47E+00 | 6.35E+00 | 4.40E+00 | 5.81E+01 | 9.29E-01 | 3.90E+00 |
| **class70** | 1.47E+00 | 5.80E+00 | - | - | 1.61E+00 | 2.04E+01 |
| **class140** | - | - | 2.48E+00 | 1.46E+01 | - | - |
| **class50** | - | - | - | - | 1.96E+00 | 4.40E+01 |
| **class150** | - | - | - | - | 3.41E+00 | 1.54E+01 |
| **class160** | - | - | - | - | 1.01E+01 | 6.30E+00 |
| **class210** | - | - | - | - | -1.84E+00 | 4.40E+00 |
| **ndvi_diff** | 2.78E+00 | 5.41E+00 | - | - | 6.58E+00 | 3.53E+01 |
| **ndvi_mean** | - | - | 3.17E+00 | 5.93E+00 | -1.40E+01 | 4.37E+01 |
| **ndvi_min** | - | - | - | - | 1.30E+01 | 3.01E+01 |
| **chor_2** | 4.96E-01 | 5.96E+00 | 5.97E-01 | 8.67E+00 | 4.56E-01 | 1.64E+01 |
| **chor_1** | 6.37E-01 | 4.05E+00 | - | - | 3.88E-01 | 9.75E+00 |
| **chor_6** | - | - | 3.73E-01 | 1.13E+01 | - | - |
| **P.tajacu** | - | - | - | - | 4.56E-01 | 8.81E+00 |
| **R.tarandus** | - | - | - | - | 1.47E+00 | 1.17E+01 |
| **M.temama** | - | - | - | - | 1.03E+00 | 7.63E+00 |
| **A.cervicapra** | - | - | - | - | 7.98E-01 | 4.31E+00 |
| **T.bairdii** | - | - | - | - | 7.19E-01 | 3.81E+00 |
| **sptrend** | 8.83E-01 | 5.92E+01 | 1.05E+00 | 6.52E+01 | - | - |
| **(Intercept)** | -2.90E+01 | 1.06E+01 | -5.23E+01 | 2.19E+01 | -5.63E+01 | 9.00E+01 |

**Table S3 (continued).** Variables included in the different tick models. *Sptrend* refers to the spatial structure. *Estimate* is the coefficient that multiplies the variable values in the logit of the multivariate logistic regression. The *Wald* parameter quantifies the relevance of the variable in the model.

|  | **Haemaphysalis longicornis** | | **Amblyomma maculatum** | |
| --- | --- | --- | --- | --- |
| **Variable** | **Estimate** | **Wald** | **Estimate** | **Wald** |
| **dist_pop** | -3.38E-05 | 19.786777 | -1.09E-05 | 11.042685 |
| **dist_road** | - | - | -9.24E-05 | 16.176154 |
| **dist_rail** | - | - | -2.08E-05 | 18.978316 |
| **hurs_max** | -1.46E-03 | 9.034179 | - | - |
| **bio10** | - | - | 0.00665109 | 4.680641 |
| **bio17** | - | - | 0.000388081 | 17.485459 |
| **bio8** | - | - | 0.001411118 | 3.598078 |
| **hurs_range** | - | - | 0.000415667 | 2.984063 |
| **class50** | 2.40E+00 | 19.219696 | -0.5904563 | 8.152572 |
| **class70** | 2.33E+00 | 9.314832 | - | - |
| **class160** | - | - | 38.90953 | 51.712888 |
| **class190** | - | - | 3.992994 | 18.291441 |
| **class140** | - | - | 1.121399 | 7.996893 |
| **class120** | - | - | -9.784858 | 15.205958 |
| **ndvi_max** | -6.75E+00 | 17.787085 | - | - |
| **ndvi_diff** | - | - | -1.375976 | 5.599242 |
| **alt** | 1.89E-03 | 28.755857 | - | - |
| **chor_2** | 6.30E-01 | 4.699658 | 1.327201 | 141.1057 |
| **chor_1** | - | - | 0.5064473 | 30.879373 |
| **chor_5** | - | - | 0.3599101 | 23.550926 |
| **sptrend** | 9.12E-01 | 184.71547 | 0.6588859 | 103.36518 |
| **(Intercept)** | 1.20E+01 | 16.804244 | -25.3395 | 7.278618 |

**Table S3 (continued).** Variables included in the different tick models. *Sptrend* refers to the spatial structure. *Estimate* is the coefficient that multiplies the variable values in the logit of the multivariate logistic regression. The *Wald* parameter quantifies the relevance of the variable in the model.

|  | **Amblyomma mixtum** | | | **Amblyomma variegatum** | | |
| --- | --- | --- | --- | --- | --- | --- |
| **Variable** | **Estimate** | **Wald** | **Estimate** | | **Wald** |  |
| **dist_road** | -5.60E-05 | 1.05E+01 | - | | - |  |
| **dist_rail** | -1.26E-05 | 2.30E+01 | - | | - |  |
| **bio8** | 1.04E-02 | 1.20E+01 | - | | - |  |
| **bio15** | 1.56E-03 | 1.05E+01 | -2.32E-02 | | 1.79E+01 |  |
| **hurs_min** | 9.81E-04 | 1.90E+01 | - | | - |  |
| **bio13** | 2.11E-04 | 4.76E+00 | - | | - |  |
| **bio7** | - | - | -1.37E-01 | | 2.97E+01 |  |
| **bio18** | - | - | 2.93E-04 | | 4.94E+00 |  |
| **class190** | 6.56E+00 | 1.20E+01 | - | | - |  |
| **class210** | - | - | -6.21E+00 | | 7.10E+00 |  |
| **ndvi_min** | 2.04E+00 | 2.83E+00 | -1.52E+01 | | 1.14E+01 |  |
| **chor_2** | 8.94E-01 | 2.48E+01 | - | | - |  |
| **chor_1** | - | - | 1.69E+00 | | 3.78E+00 |  |
| **B.tragocamelus** | 1.37E+00 | 8.13E+00 | - | | - |  |
| **A.axis** | 1.26E+00 | 7.84E+00 | - | | - |  |
| **sptrend** | 8.01E-01 | 5.24E+01 | - | | - |  |
| **(Intercept)** | -3.92E+01 | 1.94E+01 | 1.82E+01 | | 1.85E+01 |  |

**FIGURE S1 |** Distributional models showing the predicted landscape favorability for the 13 tick species that are vectors of tick-borne pathogens affecting North American cattle.


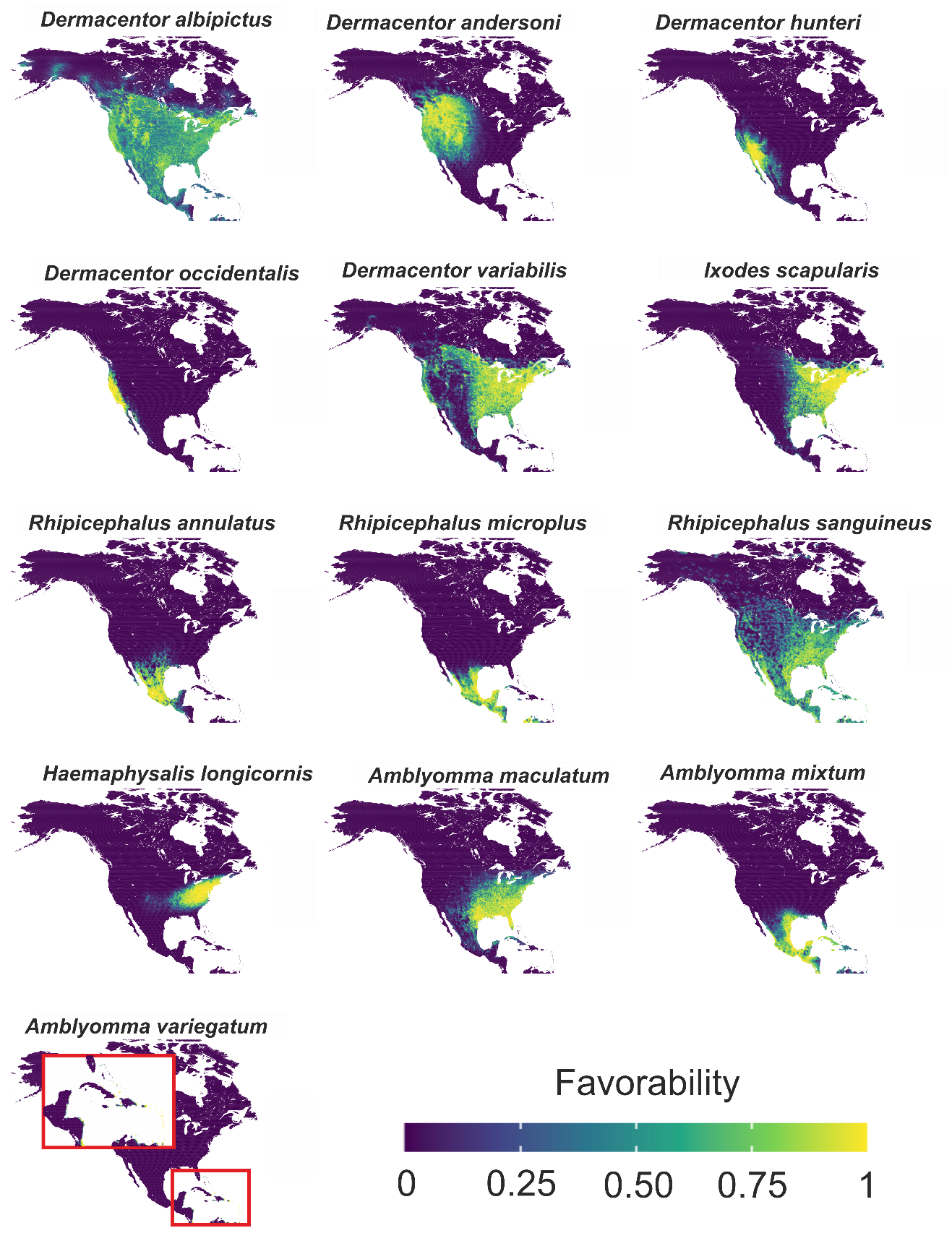


**FIGURE S2 |** Relationship between favorability values in each operational geographic unit and the number of tick records found in that unit, for each of the 13 tick species modeled.


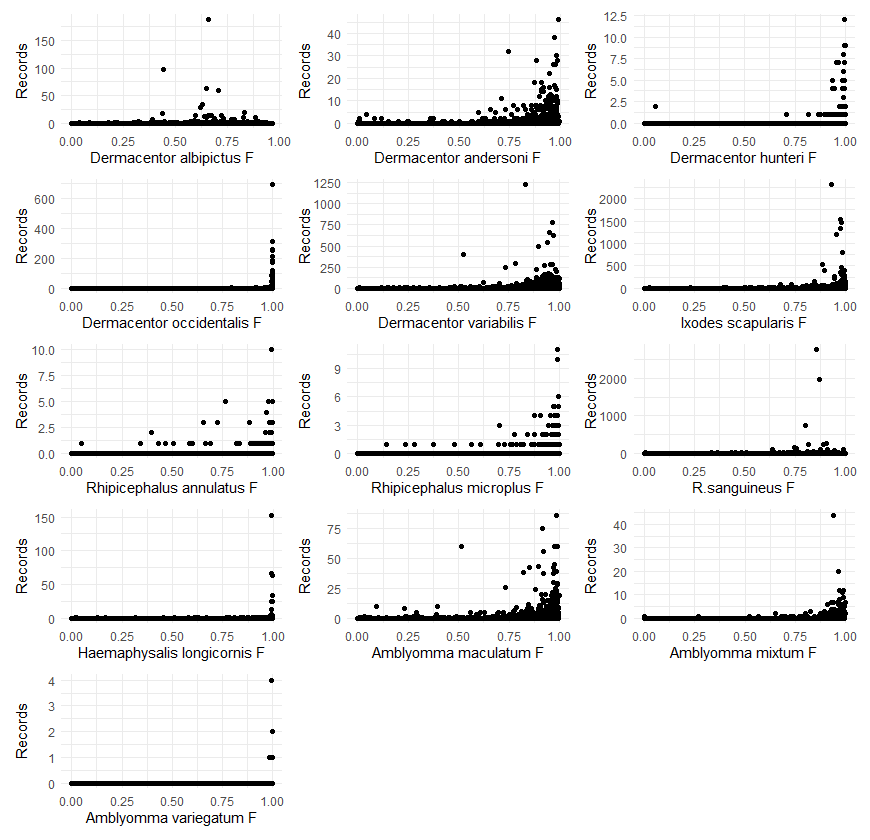


**FIGURE S3 |** Comparison of tick model performance with and without the inclusion of hosts. Positive values indicate an improvement in model performance when hosts are included, while negative values indicate a decrease in performance. CCR: correct classification rate; AUC: area under the receiver operating characteristic curve.


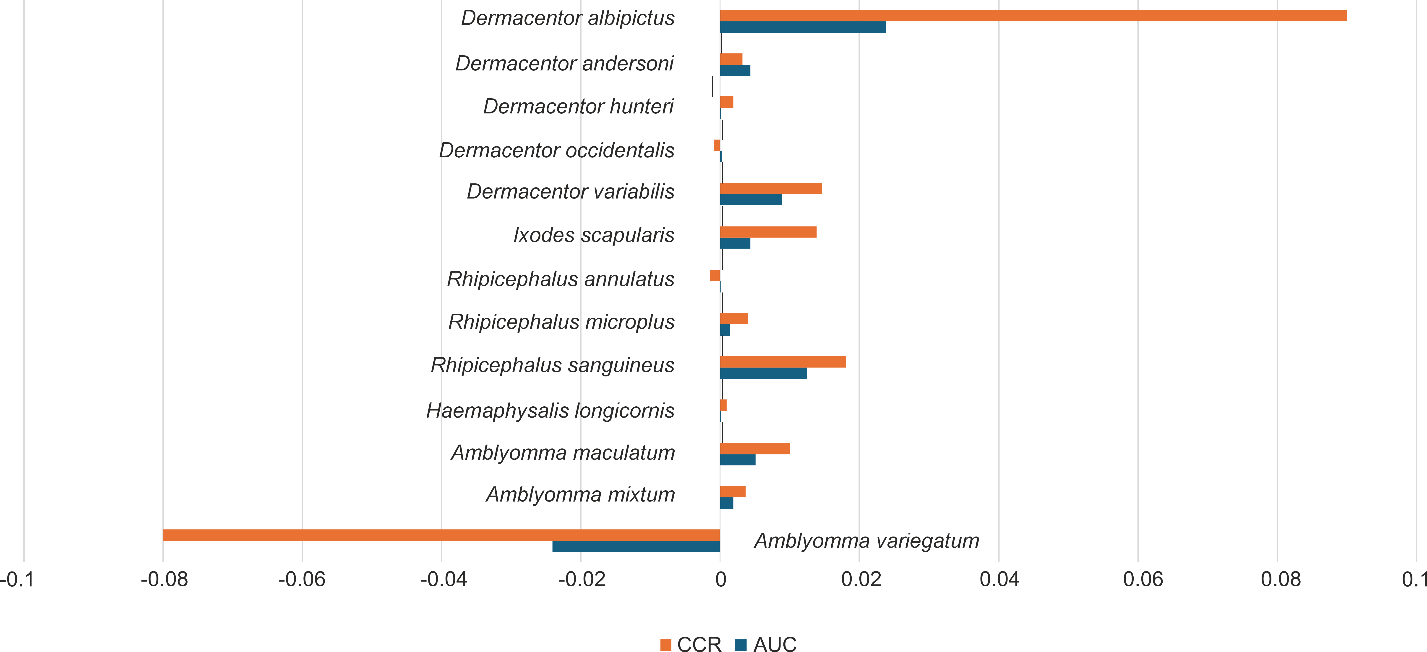


**FIGURE S4 |** Predicted risk of exposure of North American cattle to the different tick-borne diseases as well as the combination of all four diseases.

**
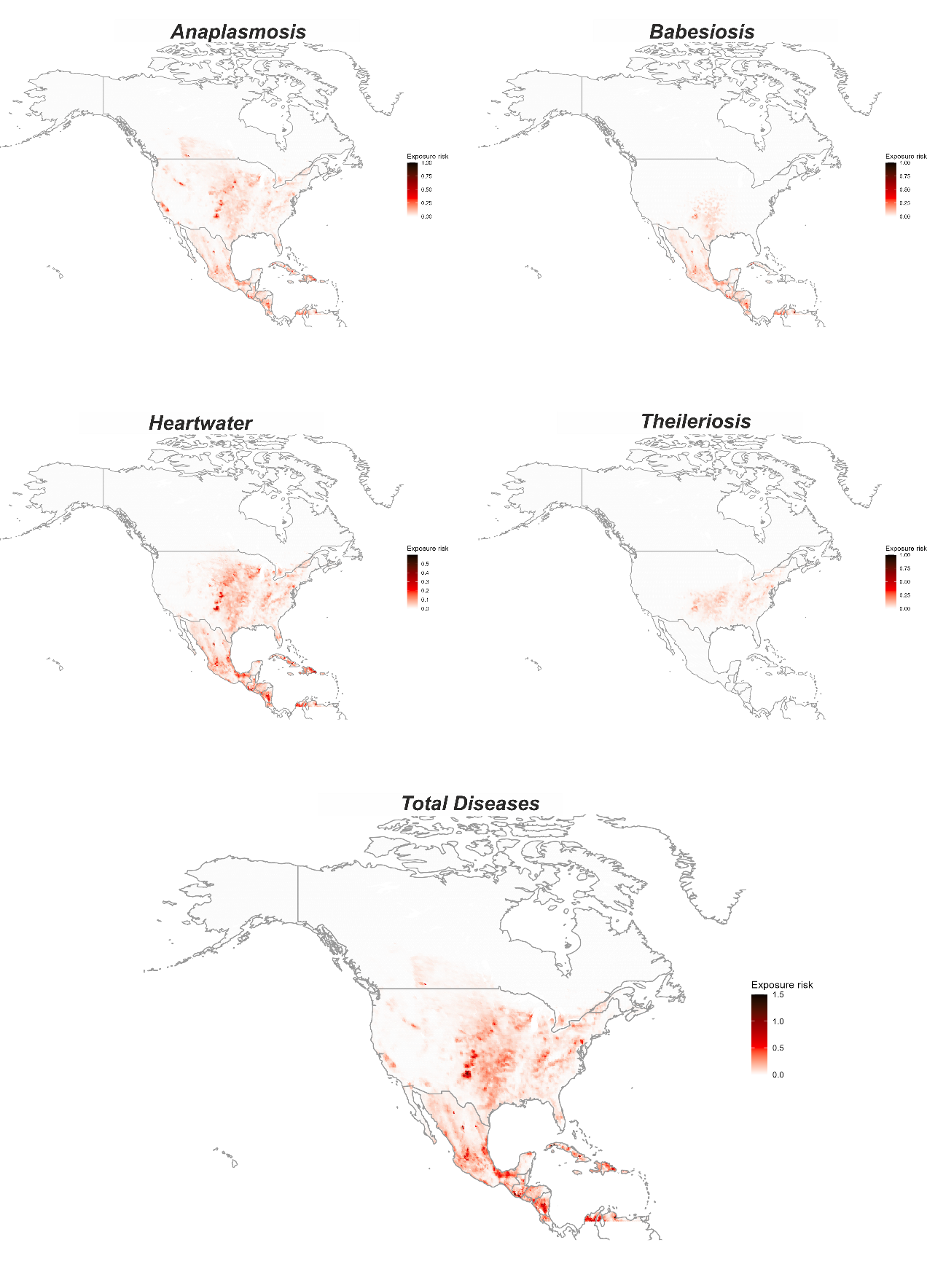
**

**FIGURE S5 |** Relationship between ungulate species diversity and tick species diversity. Each box illustrates the median, interquartile range, and potential outliers.


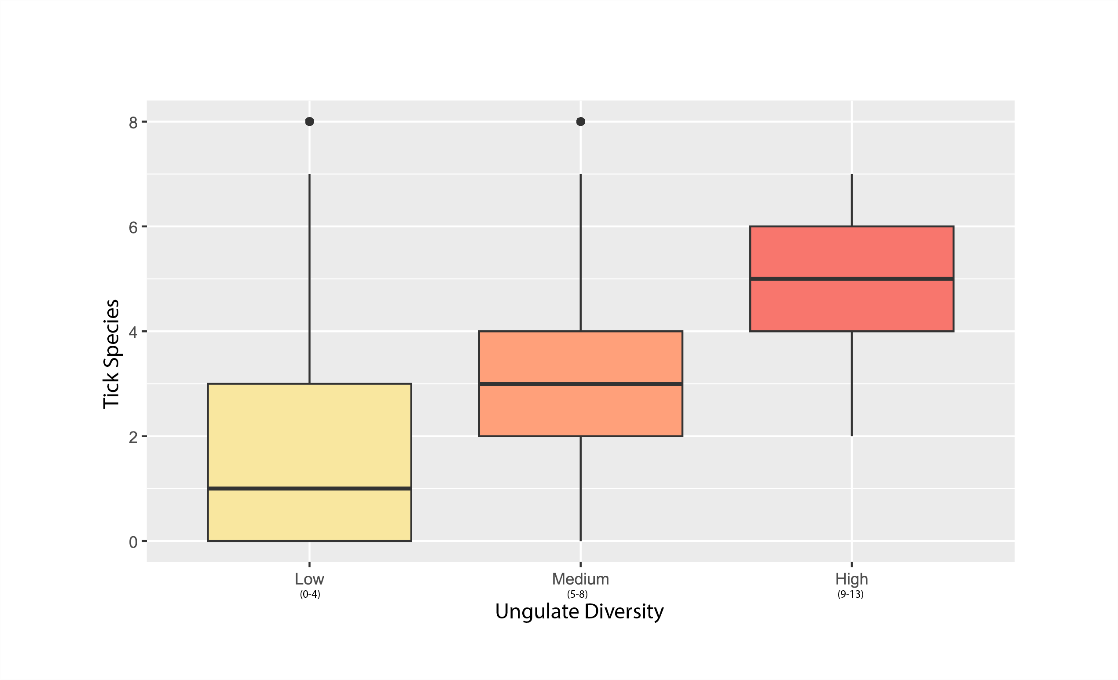


**FIGURE S6 |** Predicted risk of exposure of North American cattle to the combination of all four diseases considering ungulates and excluding them, as well as the difference between both approaches.


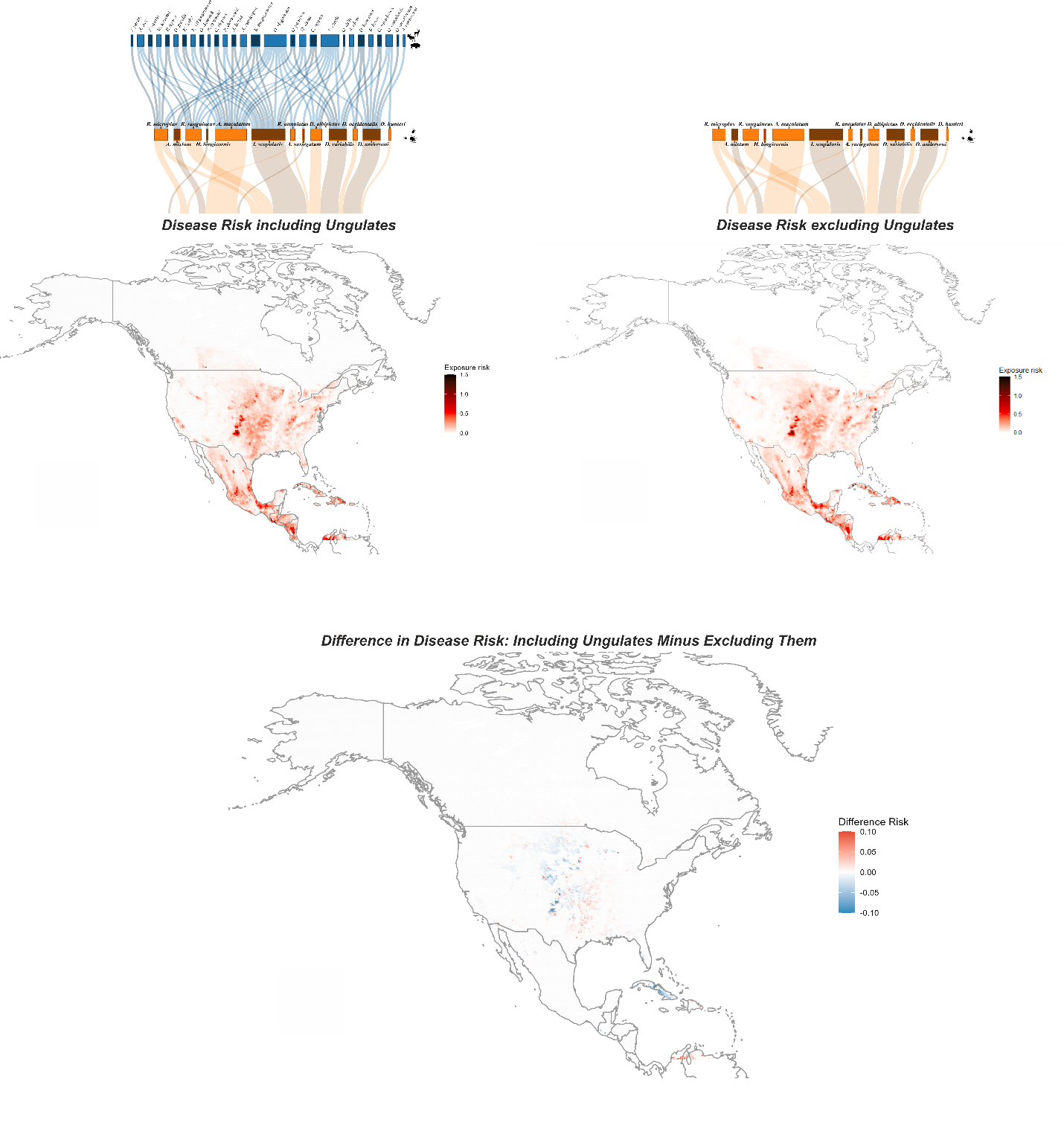

Supplement: Supplementary file 1 — Additional file1: Table S1. Explanatory variables used in the tick models. Table S2. Evaluation of the tick models. Table S3. Mathematical models for the different tick species. Figure S1. Distributional models for the different tick species. Figure S2. Favorability values of the models vs number of tick records at each grid cell. Figure S3. Tick model performance with and without the inclusion of hosts (ungulates). Figure S4. Predicted risk of exposure of North American cattle to the different tick-borne diseases as well as the combination of all four diseases. Figure S5. Relationship between ungulate species diversity and tick species diversity. Figure S6. Risk of exposure of North American cattle to tick-borne diseases considering hosts and excluding them. [file 13071_2025_7096_MOESM1_ESM.docx]
